# Potentially Inappropriate Medications in Older Adults—Prevalence, Trends and Associated Factors: A Cross-Sectional Study in Saudi Arabia

**DOI:** 10.3390/healthcare11142003

**Published:** 2023-07-12

**Authors:** Fouad F. Jabri, Yajun Liang, Tariq M. Alhawassi, Kristina Johnell, Jette Möller

**Affiliations:** 1 Department of Biostatistics, Epidemiology and Public Health, College of Medicine, Alfaisal University, P.O. Box 50927, Riyadh 11533, Saudi Arabia; 2 Department of Global Public Health, Karolinska Institutet, K9 Global Folkhälsa, K9 GPH, 171 77 Stockholm, Sweden; yajun.liang@ki.se (Y.L.); jette.moller@ki.se (J.M.); 3 Department of Clinical Pharmacy, College of Pharmacy, King Saud University, P.O. Box 2457, Riyadh 11451, Saudi Arabia; tarriq@ksu.edu.sa; 4 Medication Safety Research Chair, College of Pharmacy, King Saud University, P.O. Box 2457, Riyadh 11451, Saudi Arabia; 5 Department of Medical Epidemiology and Biostatistics, Karolinska Institutet, C8 Medicinsk Epidemiologi och Biostatistik, 171 77 Stockholm, Sweden; kristina.johnell@ki.se

**Keywords:** beers criteria, older adults, outpatients, potentially inappropriate medications

## Abstract

(1) Background: Potentially inappropriate medications (PIMs) in older adults are associated with drug-related problems, adverse health consequences, repeated hospital admissions and a higher risk of mortality. In Saudi Arabia and some Arab countries, studies of PIMs among large cohorts of older adults are limited. This study aimed to determine the prevalence of PIMs, trends and associated factors among outpatient older adults in Saudi Arabia. (2) Methods: A cross-sectional study was carried out. Over three years (2017–2019), data on 23,417 people (≥65 years) were retrieved from outpatient clinics in a tertiary hospital in Riyadh, Saudi Arabia. PIMs were assessed using the 2019 Beers Criteria. Covariates included sex, age, nationality, number of dispensed medications, and number of diagnoses. A generalized estimating equation model was used to assess trends and factors associated with PIMs. (3) Results: The prevalence of PIMs was high and varied between 57.2% and 63.6% over the study years. Compared with 2017, the prevalence of PIMs increased significantly, with adjusted odds ratios (OR) (95% confidence interval (95% CI)) of 1.23 (1.18–1.29) and 1.15 (1.10–1.21) for 2018 and 2019, respectively. Factors associated with being prescribed PIMs included ≥5 dispensed medications (OR_adjusted = 23.91, 95% CI = 21.47–26.64) and ≥5 diagnoses (OR_adjusted = 3.20, 95% CI = 2.88–3.56). Compared with females, males had a lower risk of being prescribed PIMs (OR_adjusted = 0.90, 95% CI = 0.85–0.94); (4) Conclusions: PIMs were common with an increasing trend among older adults in Saudi Arabia. A higher number of dispensed medications, increased number of diagnoses and female sex were associated with being prescribed PIMs. Recommendations on how to optimize prescriptions and implement de-prescribing strategies are urgently needed.

## 1. Introduction

With advanced age and the coexistence of two or more disorders, known as comorbidity, pharmacologic therapies increase [1]. This has been associated with the unnecessary and inappropriate use of medications among older adults [2,3,4]. There are certain medications that should rarely, if ever, be prescribed to older adults [5] and these are referred to as potentially inappropriate medications (PIMs) [6].

PIMs are defined as medications with an unfavorable risk–benefit ratio and for which more effective and safer alternatives are available [6,7]. The use of PIMs in older adults is associated with a higher prevalence of drug-related problems [8], repeated hospital admissions [9] and a higher risk of mortality [10,11], and therefore, increased health care expenditures [12,13,14]. To optimize medication use among older adults, multiple risk factors for PIMs have been identified. A higher medication count, female sex and increased health care facility visits were the most commonly identified risk factors in a study [15]. Other determinants included the patient’s age, race/ethnicity, marital status, level of education, comorbidities and availability of insurance, as well as the prescriber’s age, sex and level of experience [15].

Globally, the prevalence of PIMs in older adults has been reported as high, although it varies from 25% to 95%, depending on identification tools and study settings [16,17,18]. Using nationwide data from the United States, for example, it was estimated that the prevalence of PIMs among community-dwelling older adults was 30% [19]. In Middle East countries, studies reported a higher prevalence of PIMs among older adults. For instance, the figure was between 45% and 62% in Lebanon [20], Qatar [21] and Jordan [22]. In addition, the prevalence of PIMs among older Chinese adults increased over time from 71% in 2016 to 73.4% in 2018 [23]. Another study from Ireland showed that it increased from 39.7% to 45.6% over a five-year follow-up period [24]. Therefore, it seems that PIMs remain a worldwide problem and a major health care issue among older adults [25,26].

In Saudi Arabia, little is known about the use of PIMs and the associated risk factors in older adults. A study carried out in community medicine clinics found that 60% of the older adults included were taking at least one PIM [27]. Studies among older outpatients in Saudi Arabia showed that PIMs were associated with a higher count of medications, an increased number of patient’s diagnoses and female sex [27,28,29]. However, the small sample sizes and sampling methods used limit the generalizability of the previous findings in Saudi Arabia. In addition, little is known about the prescription trends of PIMs among older adults in Saudi Arabia, and we believe that the practice of self-medication, people’s health care-seeking behavior and other issues in the Saudi health care system, such as the lack of a national health information system, make determining the prevalence of PIMs among older adults an important area of research. Moreover, the list of PIMs changes over time based on recommendations and available research. To help with targeted interventions to reduce PIMs’ use and to be aware of older adults using PIMs and keep track of possible future adverse effects, it is therefore recommended to continuously evaluate PIMs prescriptions [30] to reduce the burden on the health care system and to improve older adults’ health. 

In this study, we aimed to determine the prevalence of PIMs among older adults (aged 65 years or above), to assess the trends in PIMs over a three-year period and to identify the associated demographic and clinical factors. 

## 2. Materials and Methods

### 2.1. Study Design, Data Source and Setting

This was a cross-sectional study conducted over three consecutive years. In 2020, we collected data for 2017, 2018 and 2019 from outpatient medical records by using the Electronic System for Integrated Health Information (E-SiHi) at King Saud University Medical City (KSUMC) in Riyadh, Saudi Arabia. E-SiHi records all visits in the outpatient clinics and includes information about patients’ demographics and clinical information. KSUMC is a large tertiary care teaching hospital with approximately 1400 inpatient beds and provides care to more than one million outpatients annually. 

### 2.2. Study Population 

The study population consisted of all adults aged 65 years or above who had at least one visit as either a new clinical appointment or a follow-up with a physician to any outpatient clinic at KSUMC from 1 January 2017 to 31 December 2019. All visits in each studied calendar year were considered. In 2017, 2018 and 2019, the number of older adults was 14,475, 16,856 and 17,395, respectively, and there were 45,629, 85,834 and 93,066 visits to the outpatient clinics during the same time period. The average number of visits per patient was 3.15, 5.09 and 5.35, in 2017, 2018 and 2019, respectively.

### 2.3. Data Extraction

Data retrieved included information on sex, age, nationality, diagnoses, names of dispensed medications and dispensing dates. The study population was split into five age-groups: 65–69 years, 70–74 years, 75–79 years, 80–84 years and ≥85 years. Nationality was dichotomized into Saudi and non-Saudi.

In the database, diagnoses were recorded by physicians. The diagnoses for each older adult were recorded for each visit in each studied calendar year according to the main groups of morbidity using the chapter levels of the International Statistical Classification of Diseases and Related Health Problems ((ICD-10)-WHO Version:2019) [31]. The number of diagnoses was categorized into; 0, 1, 2 to 4 and ≥5.

The dispensed medications were classified according to the Anatomical, Therapeutic and Chemical (ATC) classification system version 2021 [32]. The yearly number of dispensed medications was calculated for each older adult in each studied calendar year. In addition, to represent concomitant use of medications, and because the medication refill guidelines at KSUMC recommend medications be dispensed for three months at a time, the number of dispensed medications was calculated per 100-day period after the first dispensation date in each studied year. This number was then categorized into; 0, 1, 2 to 4 and ≥5.

To identify PIMs in our dataset, the list of the dispensed medications was compared with the medications that are potentially inappropriate in older adults independent of diagnosis or clinical conditions according to the American Geriatrics Society 2019 updated Beers Criteria [6]. PIMs were categorized into 0, 1, 2, 3, 4 and ≥5 in each studied calendar year. To determine the association between number of dispensed medications and PIMs, the number of dispensed medications (excluding PIMs) was categorized into; 0, 1, 2 to 4 and ≥5.

### 2.4. Statistical Analysis

A descriptive analysis was performed for the distribution of patients in strata of sex, nationality, age, number of dispensed medications and number of diagnoses. The prevalence of PIMs was presented as percentages. To account for the correlation of repeated visits by the same individual, a generalized estimating equation (GEE) model was used to assess prevalence trends. The trend was assessed in total and in subgroups by sex, nationality, age, number of dispensed medications (excluding PIMs) and number of diagnoses over the study period. Two models were performed: model 1 was a crude model, and model 2 was adjusted for sex, nationality, age, number of dispensed medications (excluding PIMs) and number of diagnoses. In addition, the associations between risk factors and PIMs were estimated using the GEE model in the aggregated data of all three years. All statistical analyses were performed in the statistical software IBM^®^ SPSS^®^ Statistics (IBM Corp. Released 2021. IBM SPSS Statistics for Windows, Version 28.0. Armonk, NY: IBM Corp).

## 3. Results

The characteristics of the study participants are shown in Table 1. Among the 23,417 older adults, the majority (91.2%) were Saudis, 51.0% were males and the mean age was 72 ± 6.7 years. Over the three years, the included study population differed slightly in the prevalence of at least one dispensed medication from 86.9% in 2017 to 87.7% in 2019. The percentages of older adults who were prescribed more than four different medications within a 100-day period following the first dispensation date increased from 47.6% in 2017 to 58.4% in 2018 and 57% in 2019.

Table 2 shows the prevalence of PIMs. While around 40% of the study population had one to two PIMs, the percentage of older adults who took three PIMs or more increased from 14.4% in 2017 to 20.9% and decreased to 18.5% in 2018 and 2019, respectively. The five most commonly prescribed PIMs were Aspirin, Pantoprazole, Levothyroxine, Insulin Glargine and Meloxicam. The results stratified by sex can be seen in Supplementary Table S1. Overall, there are only minor differences across sex. Females have slightly more medications and PIMs, and the same pattern is seen for common medications, except Aspirin, and diagnoses of diabetes and disorders of lipoprotein metabolism and other lipidaemias. 

Table 3 shows the prevalence and trend of PIMs across the study years in total and divided into subgroups by sex, nationality, age, number of dispensed medications (excluding PIMs) and number of diagnoses. Compared with the prevalence in 2017, PIMs among older adults were higher in 2018 (63.6%, OR_adjusted_ = 1.23, 95% CI = 1.18–1.29) and 2019 (60.4%, OR_adjusted_ = 1.15, 95% CI = 1.10–1.21) while taking sex, nationality, age, number of dispensed medications (excluding PIMs) and number of diagnoses into account (Table 3). We also assessed the trends for the prevalence of PIMs by sex, nationality, age, number of dispensed medications (excluding PIMs) and number of diagnoses (Table 3). The trend was similar in all subgroups except for those with less than five dispensed medications and those without any diagnosis.

The associations between various factors and PIMs are shown in Table 4. After taking into account all the covariates, a male older adult had a lower likelihood of being prescribed PIMs compared to a female older adult (OR = 0.90, 95% CI = 0.85–0.94). There was no significant association of nationality and age with PIMs. Compared to those without any other prescribed medications, having more dispensed medications (excluding PIMs) was associated with a higher OR of PIMs. The same association was found for diagnoses.

## 4. Discussion

Our study showed that PIMs were common among older adults in Saudi Arabia. We found that more than 57% of older adults were prescribed at least one PIM during a calendar year. In addition, our study found that the prevalence of PIMs increased across three years from 2017 to 2019 in almost all the subgroups by sex, nationality, age and number of diagnoses, as well as in those with five or more dispensed medications. Moreover, this study showed that older adults with a higher number of medications and/or higher number of diagnoses were more likely to be prescribed PIMs, whereas male sex was associated with lower odds of being prescribed PIMs. 

The prevalence of PIMs in this study (>57%) was generally consistent with previous studies in Saudi hospitals [29,33,34]. It seems that the problem of PIMs in Saudi Arabia is just as “serious” as in other countries, both regionally and compared to Western countries. For instance, our findings concur with other previously published results from regional studies in Kuwait [35], Qatar [21] and Jordan [22]. These studies have also reported high proportions (>50%) of outpatient older adults receiving PIMs. In addition, a recent systematic review of studies performed in several Western countries reported a weighted average PIMs of 58% among community-dwelling older adults [36]. The high prevalence of PIMs among our study population could be attributed to prescriber-related factors (e.g., prescribers do not feel responsible for changing/reviewing patients’ medications prescribed by other physicians [15] and/or limited knowledge and experience about PIMs and tools to limit such medication use). There is also a lack of geriatric medicine training in the medical schools and postgraduate programs in Saudi Arabia [37]. Moreover, a study among family and internal medicine residents toward older adult patients reported that >80% of the participants did not receive training in geriatric medicine and 87% were not willing to consider geriatric medicine as a future career [37]. This is in addition to other factors that warrant future research such as the lack of local guidelines assessing and regulating the prescription of medications among this vulnerable population. 

Not only was the prevalence of PIMs high, but we also found that it increased significantly over the study years. Similar to our results, a study in Norway also reported an increased prescription pattern of PIMs in three cross-sectional studies conducted in 1997, 2005 and 2011 [38]. PIM prevalence also increased from 50.4% to 57.5% over a ten-year follow-up period in Brazil [39]. However, some studies showed contrasting results with regards to trends in PIMs among older adults. For example, two studies in France showed decreasing trends of PIM prescriptions from 1995 to 2004 [40], and between 2011 and 2019 [41] while studies conducted in the UK [42] and in the Netherlands [43] showed that the prevalence of PIMs did not change over the investigated years. The increasing trend in PIMs in our study was parallel to an increase in the prevalence of five or more dispensed medications from around 47% in 2017 to more than 55% in 2018 and 2019. In addition, this was concurrent with the absence of any intervention, such as a pharmacist-led medication review, to improve the quality of prescribing to older adults in the study setting. Studies showed that medication reviews performed by pharmacists facilitated PIM reductions among older adults [44,45,46,47,48]. However, the outcome of interventions with the engagement of pharmacists in medication therapy reviews has not been well investigated in Saudi Arabia. 

Regarding factors associated with PIMs, the most commonly reported determinants in several studies were female sex and medication count [3,15,49,50]. Regarding sex, we found that females were more likely to be prescribed PIMs and this has also been reported in previous studies in the United States [15], France [51], Saudi Arabia [27] and other Arab countries in the region [21,22,52,53]. However, there is limited information on the potential mechanisms of the impact of sex on PIMs. Potentially, it could be explained by the association between female sex and higher use of health care services [54] and because increased health care utilization is associated with inappropriate care [55]. Therefore, further studies that focus on exploring factors that potentially make female older adults more prone to be prescribed PIMs is mandated.

The number of dispensed medications seems to be an important factor associated with PIMs [3]. Taking more medications has been reported to be associated with more PIMs among older adults in studies from countries such as the United States [15], Brazil [39], Hong Kong [45] and Saudi Arabia [27,28,29]. In addition, this association has been reported in studies that have been conducted in Arab countries that share common features with the Saudi health care system such as the United Arab Emirates [53], Qatar [21], Lebanon [52] and Jordan [22]. Our study showed that the odds of being prescribed at least one PIM was six times higher if older adults had two to four medications as opposed to not having any medication. The odds were around 24 times higher among older adults with five or more prescribed medications. The association between high medication count and PIMs could be related to the progression of diseases among such older adults and to the fact that more frequent contact with prescribers may also increase the risk of receiving PIMs. The fragmented care that older adults receive across several health care providers may also play a role [15].

In addition to the previously mentioned risk factors, and consistent with previous studies, PIMs are significantly associated with the number of different diagnoses [3,39,56]. Our study showed that the odds of being prescribed at least one PIM doubled among those patients with two to four diagnoses.

On the other hand, increasing age was not found to be a significant contributing factor for being prescribed PIMs in our study. This finding is consistent with a study conducted in the United Arab Emirates [53] although other literature regarding this factor shows different results [15]. For instance, a systematic review reported that the risk for receiving PIMs increased with advancing age [3]. In contrast, another study in the United States reported that PIMs decreased with increasing age [49]. This difference may be explained by factors related to the diversity of the PIMs’ criteria used, the study settings, the study population and the confounding effect of the number of both medications and diagnoses [57].

The strengths of our study include having repeated measurements over the three-year period, which allowed us to assess the trend of PIMs in Saudi Arabia and associated factors while considering the correlation of repeated visits of the same older adult. Moreover, it had a large sample size especially when compared to most previous studies conducted in Saudi Arabia. Furthermore, this study was based on a register of medical records which provided us with comprehensive information related to PIM prevalence and pattern. However, the study also has some limitations. First, we could not explore the underlying reasons for or medical indications of why PIMs were prescribed because these were not available in the medical records. The ability to evaluate other determinants of PIMs such as socio-economic background [15], previous hospitalization [58], educational background of the prescribers and their specialties [59] was limited due to lack of information in the electronic medical record system. Second, although using register information is a strength, we still do not know whether the patients adhere to the prescriptions and actually take the prescribed medications. Although adherence to medications is challenging for older adults [60,61], some studies have shown high levels of adherence among older adults who had PIMs [62,63]. This may aggravate the PIM problem and increase the adverse health consequences in this vulnerable population. Third, since purchasing prescription medications without a prescription and self-medication are common practices in Saudi Arabia [64,65,66,67], the overall prevalence of PIMs among older adults in the community could be underestimated, especially when older adults can acquire some PIMs such as Aspirin and other non-steroidal medications over the counter. Fourth, the cross-sectional design of this study allowed us only to study associations and not causal relationships. Finally, this study was limited to outpatient clinics; hence the prevalence of PIMs among the older adults in this study cannot be generalized to all community-dwelling older adults.

## 5. Conclusions

The prescription of PIMs was common among Saudi Arabian adults, 65 years and older, with an increasing trend. Higher count of dispensed medications, increased number of diagnoses and female sex were notable determinants. Future representative studies from community-based settings are warranted to confirm this trend and causative factors associated with PIMs prescription. In addition, recommendations and effectiveness of safer pharmacological therapies are needed.

## Figures and Tables

**Table 1:** Characteristics of older adults (≥65 years) visiting KSUMC ^a^ outpatient clinics in 2017–2019.

Characteristics	2017 (*n* = 14,475)	2018 (*n* = 16,856)	2019 (*n* = 17,395)
Gender, %			
Male	50.2	51.0	51.1
Female	49.8	49.0	48.9
Nationality, %			
Saudi	92.6	92.5	92.2
Non-Saudi	7.4	7.5	7.8
Age group (years), %			
65–69	38.7	40.3	40.9
70–74	27.4	26.7	25.7
75–79	18.8	17.8	18.4
80–84	9.5	9.6	9.5
≥85	5.6	5.6	5.5
Dispensed at least one medication, %	86.9	88.6	87.7
Yearly number of dispensed medications, Median, (interquartile range)	6.0, (2.0–9.0)	7.0, (3.0–11.0)	7.0, (2.0–11.0)
Number of dispensed medications within a 100-day period following the first dispensation date, %			
0	13.1	11.4	12.3
1	10.0	8.3	8.7
2–4	29.4	22.0	22.0
≥5	47.6	58.4	57.0
Most common dispensed medications, %			
C10AA05 (Atorvastatin)	35.8	37.7	38.0
A10BA02 (Metformin)	32.4	33.8	33.7
B01AC06 (Aspirin)	27.7	37.6	33.3
A02BC02 (Pantoprazole)	26.1	29.9	28.2
A11CC05 (Cholecalciferol)	21.9	24.7	24.6
Most common diagnoses, %			
Hypertension	42.8	40.4	37.9
Diabetes mellitus	42.2	38.9	37.0
Disorders of lipoprotein metabolism and other lipidaemias	30.4	29.7	27.4

^a^ King Saud University Medical City.

**Table 2:** The prevalence of PIMs ^a^ during 2017–2019.

	Prevalence (%)		
	2017 (*n* = 14,475)	2018 (*n* = 16,856)	2019 (*n* = 17,395)
Number of PIMs			
0	42.8	36.4	39.6
1	25.3	23.0	23.2
2	17.5	19.7	18.8
3	9.2	12.0	10.8
4	3.5	5.8	5.0
≥5	1.7	3.1	2.7

^a^ Potentially inappropriate medications.

**Table 3:** Prevalence and trend of PIMs ^a^ across the study years in total and divided into subgroups by sex, nationality, age, number of dispensed medications (excluding PIMs ^a^) and number of diagnoses.

Characteristics	Prevalence (%)			Odds Ratio (95% Confidence Interval)			
	2017 (*n* = 14,475)	2018 (*n* = 16,856)	2019 (*n* = 17,395)	2018		2019	
				Crude	Adjusted *	Crude	Adjusted *
PIMs ^a^	57.2	63.6	60.4	1.37 (1.33–1.41)	1.23 (1.18–1.29)	1.10 (1.05–1.15)	1.15 (1.10–1.21)
Sex							
Female	60.5	66.2	63.3	1.33 (1.27–1.39)	1.19 (1.12–1.26)	1.20 (1.14–1.25)	1.13 (1.06–1.20)
Male	54.0	61.1	57.6	1.41 (1.35–1.47)	1.27 (1.20–1.35)	1.25 (1.19–1.31)	1.08 (1.01–1.14)
Nationality							
Non-Saudi	58.1	67.9	61.1	1.48 (1.32–1.67)	1.45 (1.21–1.74)	1.19 (1.05–1.36)	1.16 (0.95–1.40)
Saudi	57.1	63.2	60.4	1.36 (1.32–1.40)	1.22 (1.17–1.27)	1.22 (1.18–1.26)	1.10 (1.05–1.15)
Age							
65–69	57.8	63.5	59.7	1.35 (1.29–1.42)	1.23 (1.15–1.32)	1.21 (1.14–1.27)	1.07 (0.99–1.15)
70–74	55.9	63.5	60.2	1.44 (1.36–1.53)	1.32 (1.22–1.44)	1.27 (1.19–1.35)	1.19 (1.09–1.30)
75–79	57.8	63.9	61.0	1.33 (1.24–1.43)	1.78 (1.06–1.30)	1.22 (1.13–1.31)	1.07 (0.96–1.18)
80–84	57.2	63.4	62.2	1.33 (1.21–1.48)	1.16 (1.01–1.34)	1.25 (1.12–1.39)	1.07 (0.92–1.25)
≥85	57.9	63.0	61.8	1.31 (1.15–1.49)	1.17 (0.99–1.39)	1.13 (0.99–1.30)	1.02 (0.85–1.23)
Number of dispensed medications (excluding PIMs)							
0	8.9	7.7	6.4	0.89 (0.71–1.12)	1.76 (1.39–2.23)	0.76 (0.58–0.99)	1.45 (1.14–1.86)
1	23.9	23.4	20.7	1.12 (0.95–1.32)	1.23 (0.99–1.52)	1.00 (0.84–1.19)	1.15 (0.89–1.47)
2–4	46.8	45.3	42.8	1.12 (1.03–1.19)	1.15 (1.07–1.23)	0.97 (0.90–1.05)	1.03 (0.95–1.12)
≥5	82.0	86.0	84.1	1.42 (1.34–1.50)	1.43 (1.35–1.51)	1.25 (1.18–1.33)	1.26 (1.18–1.34)
Number of diagnoses							
0	19.4	19.3	21.3	0.98 (0.83–1.17)	0.92 (0.69–1.22)	1.09 (0.92–1.29)	0.93 (0.70–1.23)
1	37.9	45.4	42.2	1.42 (1.34–1.51)	1.10 (1.02–1.17)	1.23 (1.16–1.31)	0.99 (0.93–1.07)
2–4	69.6	79.8	77.2	1.75 (1.65–1.86)	1.48 (1.39–1.58)	1.51 (1.42–1.60)	1.26 (1.17–1.34)
≥5	85.3	92.1	89.6	1.92 (1.64–2.26)	1.63 (1.38–1.93)	1.48 (1.26–1.73)	1.23 (1.05–1.45)

^a^ Potentially inappropriate medications. * Controlling for sex, nationality, age, number of dispensed medications (excluding PIMs) and diagnosis.

**Table 4:** Patient characteristics associated with PIMs ^a^ based on the aggregated data in 2017–2019, odds ratio with 95% confidence intervals.

Characteristics	Odds Ratio (95% Confidence Interval) *
Sex	
Female	Ref
Male	0.90 (0.85–0.94)
Nationality	
Non-Saudi	Ref
Saudi	0.94 (0.86–1.03)
Age	
65–69	Ref
70–74	0.96 (0.90–1.01)
75–79	0.97 (0.91–1.04)
80–84	0.96 (0.88–1.05)
≥85	1.03 (0.92–1.16)
Dispensed medications (excluding PIMs ^a^)	
0	Ref
1	2.73 (2.42–3.07)
2–4	6.28 (5.65–6.97)
≥5	23.91 (21.47–26.64)
Number of diagnoses	
0	Ref
1	1.25 (1.15–1.36)
2–4	2.12 (1.95–2.31)
≥5	3.20 (2.88–3.56)

^a^ Potentially inappropriate medications. * Generalized estimation equation regression models after controlling for sex, nationality, age, number of dispensed medications (excluding PIMs) and number of diagnoses.

## Data Availability

The data presented in this study are available upon reasonable request from the corresponding author.

## Supplementary Materials

The following supporting information can be downloaded at: https://www.mdpi.com/article/10.3390/healthcare11142003/s1, Table S1: Characteristics of older adults (≥65 years) visiting KSUMC ^a^ outpatient clinics in 2017–2019 by sex.
