# Supplementary material for: Potentially Inappropriate Medications in Older Adults—Prevalence, Trends and Associated Factors: A Cross-Sectional Study in Saudi Arabia
Source: Healthcare (Basel). 2023 Jul 12;11(14):2003. doi: 10.3390/healthcare11142003 (PMC10379671; doi:10.3390/healthcare11142003)
Supplement: Supplementary file 1 [file healthcare-11-02003-s001.zip › healthcare-2478995-supplementary.pdf]

Supplementary Table S1. Characteristics of older adults (≥65 years) visiting KSUMC [a] outpatient clinics in 2017-2019 by sex.

	Male			Female		
**Characteristics**	**2017** (n=7261)	**2018** (n=8597)	**2019** (n=8882)	**2017** (n=7213)	**2018** (n=8258)	**2019** (n=8511)
Nationality, %						
Saudi	91.8	91.4	91.2	93.4	93.6	93.3
Non-Saudi	8.2	8.6	8.8	6.6	6.4	6.7
Age group (years), %						
65–69	36.8	38.3	38.6	40.7	42.4	43.4
70–74	28.0	27.5	26.3	26.7	25.8	25.0
75–79	20.0	18.8	19.4	17.6	16.8	17.3
80–84	10.1	10.3	10.2	9.0	8.9	8.7
≥85	5.2	5.2	5.5	6.1	6.0	5.5
Dispensed at least one medication, %	85.7	87.2	86.8	88.2	90.1	88.7
Yearly number of dispensed medications, Median, (interquartile range)	5, (2-8)	6, (2-10)	6, (2-10)	6, (3-10)	8, (3-12)	8, (3-12)
Number of dispensed medications within a 100-day period following the first dispensation date, %						
0	14.3	12.8	13.2	11.8	9.9	11.3
1	10.9	8.9	9.1	9.0	7.6	8.2
2–4	31.1	22.8	23.4	27.7	21.1	20.6
≥5	43.7	55.5	54.3	51.5	61.4	59.9
Number of dispensed PIMs [b], %						
0	46.0	38.9	42.4	39.5	33.8	36.7
1	25.0	23.6	23.9	25.7	22.3	22.5
2	16.6	19.5	18.5	18.4	19.9	19.1
3	8.1	10.9	9.5	10.3	13.2	12.1
4	3.0	5.0	3.9	3.9	6.6	6.2
≥5	1.2	2.1	2.0	2.2	4.2	3.4
Most common dispensed medications, %						
Atorvastatin	34.9	36.3	37.3	36.7	39.2	38.7
Metformin	31.3	32.7	32.9	33.4	34.9	34.5
Aspirin	29.9	40.0	35.7	25.5	35.1	30.8
Pantoprazole	23.3	26.9	24.9	28.9	33.1	31.7

Cholecalciferol	13.7	15.7	16.2	30.1	34.2	33.3
Most common diagnoses, %						
Hypertension	38.5	36.4	35.2	47.1	44.5	40.7
Diabetes mellitus	42.1	38.0	37.0	42.3	39.8	37.0
Disorders of lipoprotein metabolism and other lipidaemias	26.4	25.8	24.4	34.5	33.7	30.4

[a] King Saud University Medical City

[b] Potentially inappropriate medications
